# Are we still too late? Timing of orchidopexy

**DOI:** 10.1007/s00431-022-04769-1

**Published:** 2023-01-09

**Authors:** Andrea Schmedding, Felix van Wasen, Ralf Lippert

**Affiliations:** 1 Department of Pediatric Surgery and Pediatric Urology, University Hospital, Goethe University Frankfurt, Theodor-Stern-Kai 7, Frankfurt am Main, 60590 Germany; 2 Private Practice of Pediatric Surgery, Bremen, Germany

**Keywords:** Undescended testis, Cryptorchidism, Orchidopexy, Guidelines, Pediatric surgery

## Abstract

An undescended testis is the most common genitourinary disease in boys. The German guidelines, first published in 2009, proposed the timing of orchidopexy to be before 12 months of age. The aim of the study was to analyze the implementation of these guidelines 10 years after publication. The national cumulative statistics of hospital admissions, provided by the Institute for the Remuneration System in Hospitals (InEK), and the statistics concerning procedures performed in private pediatric surgical practices of the professional association of pediatric surgeons (BNKD) regarding the time of surgeries for the year 2019 were analyzed. Data from InEK included all German hospital admissions. Data from BNKD included data from 48 private pediatric surgical practices. The hospitals treated 6476 inpatients with undescended testis, and 3255 patients were operated in private practices. Regarding the age at treatment, 15% of the hospital patients and 5% of the private practice patients were younger than 1 year and fulfilled the guideline recommendations. Forty percent of the hospital patients and 29% of the private practice patients were 1 or 2 years of age. All other patients were 3 years of age or older at the time of orchidopexy.

*  Conclusions*: The rate of orchidopexy within the first 12 months of life is remarkably low even 10 years after the publication of the guidelines. Awareness of the existing guideline must be increased for both referring pediatric and general practitioners.

**Table:** 

**What is Known:** *• In Germany, orchidopexy is performed by pediatric surgeons and urologists either in hospital settings or in private practices.* *• Most international guidelines set the age for surgical treatment of undescended testis between 12 and 18 months of age. The German guidelines, published in 2009, sets the time-limit at one year of age. Until five years after publication of the German guidelines, the number of patients treated before the first year of life was low; studies show an orchidopexy ratebetween 8% and 19% during this time.*	
**What is New:** *• This study the first to cover all administered hospital patients in Germany and a large group of patients treated in private practices. It contains the largest group of German patients with undescended testis.* *• Although almost all children participate in the routine check-up at the age of seven months, which includes investigation for undescended testis, adherence to the orchidopexy guidelines is still low. Only 15% of the hospital patients and 5% of the patients in private practice were treated before their first birthdays.*

**What is Known:**

*• In Germany, orchidopexy is performed by pediatric surgeons and urologists either in hospital settings or in private practices.*

*• Most international guidelines set the age for surgical treatment of undescended testis between 12 and 18 months of age. The German guidelines, published in 2009, sets the time-limit at one year of age. Until five years after publication of the German guidelines, the number of patients treated before the first year of life was low; studies show an orchidopexy ratebetween 8% and 19% during this time.*

**What is New:**

*• This study the first to cover all administered hospital patients in Germany and a large group of patients treated in private practices. It contains the largest group of German patients with undescended testis.*

*• Although almost all children participate in the routine check-up at the age of seven months, which includes investigation for undescended testis, adherence to the orchidopexy guidelines is still low. Only 15% of the hospital patients and 5% of the patients in private practice were treated before their first birthdays.*

## Introduction

An undescended testis is the most common genitourinary disease in boys. It is diagnosed at a rate of 1 to 4.6% at birth in full-term boys and in preterm boys, at a rate of 1.1 to 45% [1]. Testis usually descends during the first 6 months of life. After that time, spontaneous descent is rare. Secondary ascension of the testis has been described in up to 69% of the boys presenting with undescended testis [2, 3].

Males with bilateral undescended testis show lower fertility rates than men with unilateral disease [4]. The age of surgery also plays an important role in fertility rates. Several studies revealed a positive impact of early surgery regarding sperm count [5–7]. Apart from lower fertility, boys with isolated undescended testis show a nearly three times higher rate of malignancy [8]. In a Swedish study, the risk of testicular cancer was 5.4 for men who underwent orchidopexy after the age of 13, whereas the risk was 2.23 if surgery was performed prior to that age [9]. The main goal of the treatment is the preservation of fertility and a decrease in malignancy rate.

Several guidelines for the treatment of undescended testis have recently been published. Internationally, referral to a pediatric surgeon or pediatric urologist is recommended at the age of 6 months for children in whom the descent did not occur until that time or who are diagnosed after that time. The recommended age for surgery is between 6 and 18 months of age [10]. The first guidelines for the treatment of undescended testis in Germany were published in 1999 by the German Society of Pediatric Surgery. In 2009, these guidelines were revised and proposed that the time of treatment should occur before the first birthday. The two revisions in 2013 and 2016 did not change the timeline for treating this disorder [11].

Internationally, rates of orchidopexy before the first year of life range between 7 and 28% [12–14]. Before the implementation of the German guidelines in 2009, only 4% of the boys underwent surgery before the age of 1 year [15]. The impact of the guidelines indicated an increase in that rate to between 8 and 19% depending on the cohort [15–17].

The aim of our study was to evaluate the age at the time of surgery 10 years after the guidelines recommend surgery before the first birthday and 5 years after the last German cohort was analyzed.

## Materials and methods

Surgery for an undescended testis is performed either in hospitals or private practices. Procedures are mainly performed by pediatric surgeons or urologists. Hospitals allow a doctor to perform these procedures after hospital admission (inpatient), or these procedures can be done on an outpatient basis. Cumulative statistics on groups of all procedures performed in German hospitals are provided by the Federal Health Monitoring System [18]. Detailed cumulative statistics on all hospital admissions are provided by the Institute for the Remuneration System in Hospitals (InEK) [19]. The database of the InEK contains the account data of all inpatient hospital patients of Germany. They include data on diagnoses, procedures, age groups of the patients, and length of stay.

No cumulative statistics concerning outpatient procedures of hospitals or general statistics concerning procedures performed in private practices are available. Statistics on ambulatory care in private pediatric surgery practices are created by the professional association of pediatric surgeons (BNKD) [20]. They collect data of their members on an annual basis. These data include diagnoses, procedures, age groups, and for the undescended testis the side of the procedure.

In Germany, the coding of diagnoses and procedures is regulated nationally. Diagnoses have to be classified based on the International Statistical Classification of Diseases and Related Health Problems (ICD) version 10, German modification [21]. Classification of procedures must be done with the International Classification of Procedures in Medicine (OPS) [22]. The classifications are provided by the Federal Institute for Drugs and Medical Devices (BfArM). For each hospital admission, one main ICD code must be provided, and additional ICD codes can be provided if necessary. While coding of diagnoses in the hospitals is often performed by coding specialists, surgical procedures are mainly coded by surgeons.

From the database of the Federal Health Monitoring System procedures of the three groups, OPS 5–624, orchidopexy, OPS 5–625, exploration for cryptorchism, and OPS 5–626, operative procedure for abdominal testis, were analyzed regarding age groups.

The database of the InEK was analyzed for 2019. All patients under the age of 18 with the main ICD code Q53.0–9 were analyzed. Patients for whom the code for reoperation 5–983 was provided were excluded. All patients analyzed stayed at least one night at the hospital.

From the database of the BNKD, all patients who were treated during 2019 for undescended testis were analyzed. Forty-eight of the 63-member private practices provided data.

Statistics were performed using Excel®.

## Results

The statistics of the Federal Health Monitoring provided data from 2005 to 2020. The rate of procedures performed under 1 year of age increased from 7% in 2005 to 12% in 2020 (Table 1).

**Table 1:** Age groups for orchidopexy (OPS 5–624), exploration for cryptorchism (OPS 5–625), and operative procedure for abdominal testis (OPS 5–626). Numbers from [18], own presentation

	< 1 year	1–4 years	5–9 years	10–14 years	15–19 years	No. of procedures
2005	7%	44%	23%	16%	10%	10,375
2006	7%	45%	23%	16%	10%	10,134
2007	7%	44%	24%	15%	9%	10,343
2008	8%	44%	24%	15%	9%	10,909
2009	9%	45%	21%	15%	9%	11,148
2010	10%	44%	22%	15%	8%	11,503
2011	10%	43%	21%	17%	9%	11,659
2012	11%	42%	20%	17%	9%	11,617
2013	11%	41%	20%	18%	10%	11,186
2014	10%	41%	20%	18%	10%	11,251
2015	11%	42%	20%	17%	10%	11,625
2016	12%	42%	20%	16%	10%	11,948
2017	12%	43%	19%	16%	10%	11,964
2018	11%	44%	18%	17%	10%	11,778
2019	11%	42%	18%	18%	11%	11,642
2020	12%	40%	18%	19%	12%	10,239

### Detailed data for 2019

In 2019, 6476 children with undescended testis underwent orchidopexy inpatient at a hospital. The mean length of stay was 1.5 days. In 58%, unilateral undescended testis was the main cause of admission, in 31% bilateral undescended testis, in 8.3% not further specified, and in 3.4% ectopia testis (Table 2).

**Table 2:** Main diagnoses in children with undescended testis

Diagnosis	Hospital patients		Patients in outpatient practice	
	Number	%	Number	%
Undescended testis, unilateral	3738	58%	2639	81%
Undescended testis, bilateral	1986	31%	562	17%
Undescended testis, not further described	534	8.3%	63	1.9%
Ectopic testis	218	3.4%		

Forty-eight private practices provided data for 2019. In these practices, 3255 patients with undescended testis underwent surgery. In 81%, a unilateral undescended testis was treated, of these 51% were on the left side, 49% were on the right side, 17% had bilateral undescended testis, and in 1.9%, it was not specified.

Procedures for undescended testis consisted mainly of orchidopexy with standard inguinal approach (OPS 5–530.02 and 5–624.4) in 90% of the hospital patients and 93% of the patients in private practices. In 6.4% of the hospital patients and 1.2% of private practice patients, laparoscopic exploration or laparoscopic replacement of abdominal testis was performed (Table 3).

**Table 3:** Number of patients undergoing procedures for undescended testis

OPS	Procedure	Hospital patients		Patients in outpatient practice	
		Number	%	Number	%
5–622	Removal of testis	201	3.1%	33	1.0%
5–530.02	Closure of inguinal hernia with orchidopexy with a standard inguinal approach	638	9.9%		
5–624.4	Orchidopexy with a standard inguinal approach	5206	80%	3024	93%
5–624.5	Orchidopexy scrotal	381	5.9%	63	1.9%
5–624.x/y	Orchidopexy, other	53	0.8%	27	0.8%
5–625.4	Cryptorchidism, exploration inguinal	85	1.3%	5	0.2%
5–625.5	Cryptorchidism, exploration abdominal, open surgery	9	0.1%		
5–625.6	Cryptorchidism, exploration abdominal, laparoscopic	228	3.5%	21	0.6%
5–626.0	Surgical replacement of abdominal testis in the scrotum, without microvascular anastomosis, open surgery	148	2.3%	63	1.9%
5–626.2	Surgical replacement of abdominal testis in the scrotum, without microvascular anastomosis, laparoscopic	191	2.9%	18	0.6%
5–626.x	Surgical replacement of abdominal testis in the scrotum, others	19	0.3%		

Additional procedures were documented for the hospital patients. Biopsies were taken in 14 of them (0.2%), and inguinal hernia repair was done in 21%. In four patients, reconstruction of the ductus deferens was documented. The mean length of stay was 1.5 days.

**Fig. 1:**
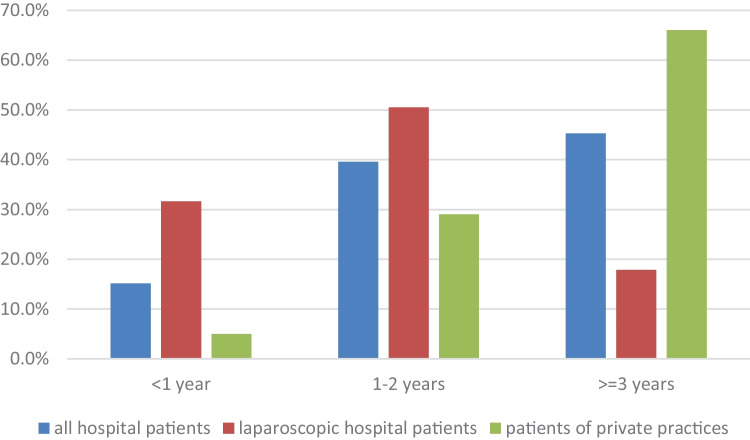
Age at which surgery was performed in German hospitals for undescended testis in all patients (*n* = 6475), patients undergoing laparoscopic procedures (*n* = 392), and patients treated in private practice with age provided (*n* = 3139)

Of the hospital patients, 15% were admitted under the age of 1, 40% were between 1 and 2 years of age, 22% between 3 and 5 years of age, 14% between 6 and 9 years of age, and 9% more than 10 years of age. Of those patients who underwent laparoscopic surgery, 32% were under the age of 1 year, 51% were between 1 and 2 years of age, 10% between 3 and 5 years of age, 4% between 6 and 9 years of age, and 4% more than 10 years of age. Of the patients from private practices, 5% were under the age of 1 year, 29% under the age of 2 years of age, and 66% more 3 years of age; in 4%, the age was unknown (Fig. 1).

## Discussion

Several studies have shown the benefits of early orchidopexy in boys. Boys had significant recovery of testicular volume when surgery was performed before the age of 2 [23]. Feyles reported that in the group of patients who underwent surgery during the first year of life, significantly more patients had normal sperm count and motility in relation to those who underwent surgery later [7]. These results led to early orchidopexy recommendations by international guidelines. The American Urological Association recommended orchidopexy for boys with undescended testis at the age of 6 months to within 1 year [24]. The European Association of Urology stated that scrotal positioned testis should be achieved at the age of 1 year with the latest by 18 months [25]. The German guidelines on the timing of orchidopexy at the age of 1 year were published in 2009 and updated in 2016 [11].

In Germany, screening of healthy boys for undescended testis is performed by pediatricians or general practitioners. Until the age of 6 months, there are five obligatory check-ups for infants. All include an examination of the genitalia. The fifth check-up (U5), which has to be undertaken between the sixth and seventh month of life, includes the explicit check for undescended testis [26]. As almost all of the children in Germany participate in the U5 [27], primary undescended testis should be detected up to the seventh months of life. Despite this early check-up numbers of procedures for orchidopexy, cryptorchism and abdominal testis were 10% in 2010 and went up to 12% in 2020. As these data also contain procedures performed for other conditions than undescended testis, we also analyzed the database of InEK, which provides information on the procedure together with the diagnosis. Ten years after the publication of the German guidelines, our data showed that orchidopexy was performed in the first year of life only in a minority of boys (15% of hospital patients and 5% of patients of private practices). In the subgroup of patients who underwent laparoscopy for exploration of cryptorchidism or for treatment of abdominal testes, 33% underwent surgery before the first year of life. Even when presuming that abdominal or not-palpable testis is seen in primary undescended testis, the numbers were higher than the whole group but still much lower than they should have been.

Comparing our data with the international literature showed that rates in Germany were much lower than in several other countries where the rate of orchidopexy performed before 1 year of life was up to 40% (Table 4). Reasons for the delayed timing of orchidopexy in Germany had been addressed in previous studies. In a nationwide survey among German pediatricians, 73% considered the referral of the primary care pediatrician as the most important factor for the timing of surgery [28]. This is supported by the fact that in general patients have to be referred to a surgeon by a pediatrician or general practitioner for treatment of undescended testis. Only the minority of private insured patients can be treated without a referral. Knowledge of general practitioners and outpatient pediatricians regarding undescended testis was important [28] but does not automatically lead to early referral [29]. When such information is presented in an adequate format, it can have a great benefit. In a setting in New Zealand, the implementation of electronically available guidelines led to an increase in the rate of surgeries in the first year of life from 16% in 2012 to 39% in 2018 [30].

**Table 4:** Rate of surgery before 1 year of life

Country	Period	Rate of orchidopexy performed before 1 year of life	Data source
China [12]	2012–2019	7%	One medical center
Sweden [31]	2011	8%	Administrative data, age 0–5 years
Ontario, Canada [31]	2011	10%	Administrative data, age 0–5 years
Israel [13]	2003–2013	11%	One medical center
England [31]	2011	12%	Administrative data, age 0–5 years
**Germany – our data**	**2019–2021**	**5–15%**	**Administrative data, age 0–17 years**
Finland [31]	2011	16%	Administrative data, age 0–5 years
Singapore [32]	2007–2011	23%	One medical center
Scotland [31]	2011	28%	Administrative data, age 0–5 years
Bosnia-Herzogovina [14]	2008–2010 2015–2017	29%	One medical center
Victoria, Australia [31]	2013	29%	Administrative data, age 0–5 years
New Zealand [30]	2018	39%	One medical center
Iceland [31]	2011	40%	Administrative data, age 0–5 years

Access to surgery and the type of hospital in which the child is treated also played a role for timing. Despite early referral, waiting for surgery can result in a delay in treatment [33]. In Germany, the rate of early orchidopexy was significantly higher in hospitals with pediatric surgical departments than without [28].

In the literature, other factors for delayed surgical treatment were the kind of health insurance [34], socioeconomic status, and distance to the treating center [35]. In a study from Sweden, high income and absence of social security support but not the level of education or patient migration status were associated with a higher rate of early orchidopexy [35]. While health insurance had an impact on this rate in the USA [34], insurance did not play a role in Israel [13].

Our analysis shows the dilemma of existing guidelines on the one hand and actual implementation in medical routine. Guidelines are published online [11]. A summary of the guideline of undescended testis was published twice at the journal of the German Society of Pediatrics and Adolescent Medicine (DGKJ) [36, 37]. Press releases can be a means to distribute information, but for undescended testis, it was used sparsely so far. There were two press releases by the German Society of Urology between 2010 and 2015 [38], one by the German Society of Pediatric Surgery in 2014 [39], and none by the DGKJ. Because almost all children get their check-up by a pediatrician or general practitioner in time, but they receive surgery after the recommended time, efforts should be taken by the relevant medical societies to address this problem to the target group of primary care practitioners and the surgical partners.

### Strength and limitations

Our study consisted of two large cohorts of data from admitted hospital patients in Germany and data from pediatric surgical outpatient practices. The strength of our study lies in the large size of the cohort and the inclusion of urological and pediatric surgical patients. For hospital-admitted patients, data were obtained for the whole cohort, so no bias in any sample was found. Unfortunately, these numbers do not reflect all patients in Germany as there are two more settings in which boys with undescended testes are operated: (1) outpatient surgeries of hospitals and (2) outpatient practices other than pediatric surgical ones. For these settings, no German-wide statistics were available. This lack of data is the main limitation of the study.

The impact of the rate of secondary ascended testis on the timing of orchidopexy could not be analyzed in our data as administrative claims data do not provide clinical information. In a study from Korea, acquired cryptorchidism was seen as a reason for orchidopexy after the age of 5 years in 13% of the cases [40]. A study from Denmark reported ascensus testis accounting for 58% of all cases of undescended testis at the age of 18 months and 69% after the age of 36 months [3].

## Conclusion

The rate of orchidopexy within the first 12 months of life is still low 10 years after the publication of the guidelines. As pediatric surgeons and urologists rely on patient referral by general practitioners and pediatricians, the awareness for the pathology and information on the actual guideline should be increased in this group.

## Data Availability

Data availability statement:The datasets of hospital data analysed during the current study are available of the Federal Health Monitoring System [18] and the Institute for the Remuneration System in Hospitals [19]. The dataset of the private practices analysed during the current study are available from the BNKD (info@kinderchirurgie.com) on reasonable request.
